# Golgi phosphoprotein 3 promotes angiogenesis and sorafenib resistance in hepatocellular carcinoma via upregulating exosomal miR-494-3p

**DOI:** 10.1186/s12935-022-02462-9

**Published:** 2022-01-24

**Authors:** Ying Gao, Zheng Yin, Yunling Qi, Hong Peng, Wenbin Ma, Ruizhi Wang, Wen Li

**Affiliations:** 1 Laboratory of General Surgery, The First Affiliated Hospital, Sun Yat-sen University, Guangzhou, 510080 China; 2 Division of Vascular Surgery, National Guangdong Joint Engineering Laboratory for Diagnosis and Treatment of Vascular Diseases, The First Affiliated Hospital, Sun Yat-sen University, Guangzhou, 510080 China; 3 Center of hepato-pancreatobiliary surgery, The First Affiliated Hospital, Sun Yat-sen University, Guangzhou, 510080 China; 4 School of Life Sciences, Sun Yat-sen University, Guangzhou, 510275 China; 5 Department of Laboratory Medicine, The First Affiliated Hospital, Sun Yat-sen University, Guangzhou, 510080 China

**Keywords:** GOLPH3, Hepatocellular carcinoma, Exosomal miR-494-3p, Angiogenesis, Sorafenib resistance

## Abstract

**Background:**

Golgi phosphoprotein 3 (GOLPH3) has been frequently reported as an oncoprotein in a variety of tumors. However, its role in the cancer-associated intercellular signaling communication has not yet been explored. This study aimed at exploring whether GOLPH3 regulates angiogenesis and sorafenib resistance via exosomal mechanisms in hepatocellular carcinoma (HCC).

**Methods:**

In vivo assays were performed to elucidate the function of GOLPH3 in HCC. Exosomes of HCC cells were isolated by differential centrifugation, and then measured and quantified using nanoparticle tracking analysis (NTA), BCA assay, western blot (WB), and transmission electron microscopy (TEM). Differentially expressed miRNAs in exosome were analyzed and verified through small RNA sequencing (sRNA-seq) and reverse-transcription polymerase chain reaction (RT-PCR). In addition, a series of in vitro assays were performed to determine the function of exosomes and miR-494-3p in HCC. The candidate target gene of miR-494-3p was identified by bioinformatics prediction and dual-luciferase reporter assay.

**Results:**

Downregulation of GOLPH3 expression could suppress angiogenesis and enhance sorafenib sensitivity in HCC. Exosomes derived from GOLPH3 overexpression HCC cells promoted the angiogenesis ability of HUVECs and induced sorafenib resistance in HCC cells. A total of 13 differentially expressed miRNAs between negative control and GOLPH3 knockdown group were found in exosomes. However, GOLPH3 was only associated with miR-494-3p expression level in exosomes derived from HCC cells without affecting total cellular miR-494-3p content. Results confirmed that exosomal miR-494-3p promotes angiogenesis of HUVECs and sorafenib resistance in HCC cells through directly targeting PTEN.

**Conclusions:**

HCC cells with high expression levels of GOLPH3 could promote angiogenesis and sorafenib resistance by enhancing exosomal miR-494-3p secretion to recipient HUVECs and HCC cells, respectively.

## Background

Hepatocellular carcinoma (HCC) is one of the most common malignant tumors with high morbidity and mortality worldwide [1–3]. Most HCC patients are diagnosed at an advanced stage, leading to poor prognosis with an average median survival time of 6 months and an overall 5-year survival rate of less than 15% [4]. Sorafenib is a multikinase inhibitor targeting vascular endothelial growth factor (VEGF)-induced angiogenesis. Sorafenib exhibits significant efficacy to placebo in improving overall survival (OS) of advanced HCC patients [5]. In the past 10 years, sorafenib has been the only available conventional therapy for advanced-stage HCC [4, 6]. Notably, several advanced patients are either insensitive to sorafenib or present with drug resistance after sorafenib treatment, following rapid progression of disease [5]. Therefore, studies should explore potential strategies to enhance efficacy of anti-angiogenic therapy and reduce sorafenib resistance thus improving clinical outcome of advance HCC patients.

Golgi phosphoprotein 3 (GOLPH3) is a matrix protein located on the opposite sides of Golgi apparatus. GOLPH3 plays an important role in maintenance of Golgi banding structure, vesicle transport, protein classification and protein glycation [7–9]. Recent studies report that GOLPH3 is implicated in progression of various cancers and affects disease prognosis [8, 10–12]. Overexpression of GOLPH3 promotes angiogenesis by activating and maintaining NF-κB pathway, which is modulates recurrence, metastasis and prognosis of HCC [13]. In bladder cancer, GOLPH3 mediates gemcitabine and cisplatin chemoresistance by promoting cancer stem cell phenotypes [14]. However, the role and mechanism of GOLPH3 on modulating cancer-associated intercellular signaling communication to promote angiogenesis and resistance of sorafenib has not yet been explored.

Exosomes are small intracellular membrane-based vesicles with different compositions and play various roles in biological and pathological processes [15]. Exosome secretion is a characteristic feature of malignancy. Tumor-derived exosomes modulate tumor growth, angiogenesis, immune escape, and therapeutic resistance by transporting biomolecules such as microRNAs or proteins into the tumor milieu [16, 17]. To data, the effect of GOLPH3 on the number and composition of biomolecules in HCC cell-derived exosomes had not been elucidated.

The findings of the present study showed that downregulation of GOLPH3 expression in HCC cells suppressed angiogenesis and enhanced sorafenib sensitivity in vivo. Exosomes derived from HCC cells overexpressing GOLPH3 promoted angiogenesis and sorafenib resistance and the quantity of exosomes was not altered by GOLPH3 overexpression. Small RNA sequencing and RT-PCR analysis showed that upregulation of GOLPH3 increases exosomal miR-494-3p expression level. Notably, exosomal miR-494-3p promoted angiogenesis and sorafenib resistance by inhibiting PTEN expression in HUVEC and HCC cell lines. These results indicated that GOLPH3 exerted its functions by upregulating expression of miR-494-3p in exosomes derived from HCC cells. This is the first study to report that GOLPH3 regulates angiogenesis and sorafenib resistance through exosome mechanism. The findings indicate that targeting GOLPH3 and its novel exosomal miR-494-3p pathway can provide a new therapeutic approach for HCC treatment.

## Methods

### Cell lines and cell culture

Eight human HCC cell lines (MHCC-97H, HCCLM3, SNU-449, Huh7, SK-hep-1, PLC/PRF/5, Hep3B and HepG2) and one immortalized hepatocyte cell line (LO2) were acquired from the Shanghai Cell Bank at the Chinese Academy of Sciences. Human umbilical vein endothelial cells (HUVECs) and human embryonic kidney (HEK293T) cell line from ATCC. HCC cell lines and HEK293T were cultured in Dulbecco’s modified Eagle’s medium (DMEM) (#C11995500BT, Gibco, MA, USA), supplemented with 10% fetal bovine serum (FBS) (#ST30-3302, PAN-Biotech, Germany) and 1% Penicillin–Streptomycin (#15140-122, Gibco, MA, USA). EBM-2 Basal Medium containing EGM-2 SingleQuot Kit Supple&Growth Factor (#CC-3156 & CC-4176, Lonza, USA) was used to culture HUVECs. Cells were cultured at 37 °C in an incubator (Thermo Fisher Scientific, Waltham, MA, USA) with 5% CO_2_ under humidified atmosphere. Cells were cultured in DMEM with exosome-free FBS for 48 h for prepare the culture medium for exosome isolation.

### Construction of the recombinant lentiviral vector and stable cell lines

All plasmids were constructed by Genecreate Company (Wuhan, China). The plasmid pLKO.1-puro (#8453, Addgene, MA, USA) was used to construct the GOLPH3 shRNA lentiviral expression vector. The oligonucleotides for human GOLPH3 were 5′-AAGGTAATCTGTAAGTCAGAT-3′(sh1) and 5′-TGGAATCCATTAAAATTG.

CATTA-3′(sh2). GOLPH3(NM_022130) complementary DNA (896 bp) was cloned into pCDH-CMV-MCS-EF1-copGFP (#CD511B-1, System Biosciences, Palo Alto, CA, USA) plasmid.

Recombinant lentivirus was generated by transiently co-transfecting HEK293T cells with lentiviral plasmid along with the ecotropic packaging plasmids [Target vector: psPAX2 (#12260, Addgene, MA, USA): pMD2.G (#12259, Addgene, MA, USA)  =  4:3:1] using lipofectamine 2000 (Invitrogen, Carlsbad, CA, USA). Forty-eight hours after transfection, the supernatant containing lentiviral particles was passed through a 0.45-μm filter and stored at – 80 °C until further analysis. Target cells were maintained in the collected viral supernatant supplemented with 8 μg/mL polybrene (#TR-1003, Sigma, St. Louis, MO, USA) for 12 h. Next, the supernatant was replaced with fresh DMEM medium supplemented with 10% FBS, followed by selection of the stable lines. For GOLPH3 knockdown, the shRNA-infected cells were allowed to grow for 48–72 h, after which they were selected with 2 μg/mL puromycin (#ant-pr-1, Invitrogen, Carlsbad, CA, USA) for 2 weeks. The final stable knockdown cell lines were maintained in a medium containing 0.5 μg/mL puromycin in subsequent cultures. For GOLPH3 overexpression, the GOLPH3-expressing lentivirus transfected cells co-expressing GFP were selected using flow cytometry (BD Biosciences, USA).

### Immunoblotting

Cells or exosome pellets were lysed using RIPA lysis and extraction buffer (#89900, Thermo Fisher Scientific, USA) containing protease and phosphatase inhibitor mixture (#KGP602 & KGP603, KeyGEN, China) on ice for 30 min. Cell lysates were centrifuged at 12,000×*g* for 15 min. Denatured protein samples (10–20 μg) were separated by SDS-PAGE gel and the proteins were transferred to polyvinylidene difluoride (PVDF) membranes (Merck Millipore, USA). Membranes were then blocked with 5% bovine serum albumin (BSA) dissolved in TBS containing 0.1% Tween 20 (TBST) and incubated for 1 h at room temperature (RT). Blots were further incubated with rabbit polyclonal anti-GOLPH3 (Abcam, ab236296,1:1000), anti-PDCD6IP (Alix, ProteinTech; #10427-2-AP,1:1000), anti-CD63 (ProteinTech; #25682-1-AP,1:1000), mouse monoclonal anti-Bcl-2 (Cell signaling Technology, #15071S,1:1000), anti-Bax (Cell signaling Technology, #89477S,1:1000), or anti-GAPDH (glyceraldehyde-3-phosphate dehydrogenase; ProteinTech, #60004-1-AP,1:5000) at 4 °C overnight. Blots were then washed and incubated with goat anti-rabbit (Cell signaling Technology, #7074,1:5000) or mouse (Cell signaling Technology, #7076,1:5000) secondary antibodies for 1 h at RT. Western blot images were visualized using a Tanon 5200 Multi chemiluminescent imaging system (Tanon).

### RNA isolation and real-time quantitative PCR (qRT-PCR)

Total RNA was extracted using TRIzol reagent (#15596018, Invitrogen, USA). RNA was reverse transcribed to obtain cDNA using the PrimeScript™ RT Reagent Kit (#RR037A, Takara) according the manufacturer’s instructions. RNA purity and concentration were determined using a NanoDrop 2000 (Thermo Fisher Scientific). High-quality RNA samples with A260/A280  > 1.8 and A260/A230  > 2.0, were used for subsequent experiments. Relative expression of mRNA of target genes was determined by real-time quantitative PCR using SYBR^®^ Green master mix (#4887352001, Roche, Basel, Switzerland). miDETECTTM miRNA External Control (#miRB0000010, Ribobio, Guangzhou, China) was added to the RNA extraction lysate for miRNA expression analysis using HCC exosomal samples. cDNA was synthesized and qRT-PCR of miRNA was performed using miDETECT A Track miRNA qRT-PCR Starter Kit (#C10712-1, Ribobio, Guangzhou, China). qRT-PCR was performed using LightCycler^®^ 480II system (Roche, Basel, Switzerland). Analysis was performed for 40–45 cycles with three biological samples. The following mRNA primer pairs were used: GOLPH3, sense (5′-CTCCAGAAACGGTCCAGA-3′), antisense (5′-CCACCAGGTTTTTAGCTAATCG-3′); GAPDH, sense (5′-CTGACTTCAACAGCGACACC-3′), antisense (5′-TGCTGTAGCCAAATTCGTTG-3′). miRNA primer pairs were purchased from Ribobio company (#miRA1000342, miRA1000688, miRA0000010, miRA0002816, miRA1000251, miRA1000351, miRA1000805& miRA1001268, Guangzhou, China). Expression levels in cell samples were evaluated relative to mRNA or miRNA expression levels of the internal control (GAPDH or U6 small non-coding RNA) using the 2^−ΔΔCt^ method. External control miRNA was used for relative quantitative analysis in exosome samples.

### Animal experiments

Five-week-old Balb/c nude mice were purchased from Vital River Laboratory Animal Technology Company (Beijing, China). Mice were randomly assigned to four groups (n  = 5): MHCC-97H shC, MHCC-97H sh1, MHCC-97H shC  +  sorafenib, MHCC-97H sh1  +  sorafenib. Equal amounts of MHCC-97H shC or MHCC-97H sh1 cells (1 × 10^7^) were subcutaneously injected into the flank of each mouse and tumor volume determined. Tumor volume was calculated using the formula: volume  =  ab^2^/2 (a, the largest diameter; b, the perpendicular diameter). Drug administration was performed when the tumors reached a volume of ≈ 200 mm^3^. Mice received equal volume of sorafenib (15 mg/kg, #S7397, Selleck, Shanghai, China) or vehicle [DMSO (#196055, Biyuntian, Shanghai, China)] through intragastric administration every day for 3 weeks or less until the tumors reached 2000 mm^3^. Animals were sacrificed by cervical dislocation and the tumors excised and weighed. All animal procedures were performed in accordance with the guidelines of the Laboratory Animal Ethics Committee of Sun Yat-sen University.

### Immunohistochemistry (IHC) analysis

Mouse tumor tissues were fixed in formalin and embedded in paraffin. Paraffin-embedded sections used for IHC staining were deparaffinized and rehydrated. Antigen retrieval was performed on sections and endogenous peroxidase was using quenched the further blocked using endogenous peroxidase. Sections were incubated in citrate buffer solution (pH 6.0) and boiled for 20 min to retrieve antigens. Subsequently, sections were incubated with anti-GOLPH3 (ab98023, abcam, 1:500) or anti-CD34 (#380824, ZenBio, 1:200) overnight at 4 °C. Immunostaining was conducted using the Envision System with diaminobenzidine (DAB) (#K406511-2, Dako, Glostrup, Denmark). PBS was used instead of primary antibody in the negative control group. Immunoreactivity for GOLPH3 was scored using a semiquantitative method based on the cellularity and intensity of the expression. Microvessel density (MVD) in tumor tissues was evaluated using a previously described method [18]. Briefly, the microvessel density (MVD) in xenograft tumor tissues was evaluated based on CD34 staining, and was quantified in three most intensely vascularized areas in each vascularized area or the whole area of a small tumor under light microscopy at a magnification of 200 ×. Any discrete cluster or single cell stained for CD34 was counted as one microvessel. The average value of the vessel count per field for each case was used as the final MVD value.

### Exosome isolation

Exosomes were isolated through standard centrifugation steps, as previously described [19]. Briefly, the culture medium was centrifuged at 300×*g* for 10 min to remove detached cells. The resulting solution was centrifuged at 2000×*g* for 20 min, followed by centrifugation at 10,000×*g* for 45 min. The supernatant was then filtered through a 0.22 μM filter mesh (Merck Millipore, USA). The filtrate was ultracentrifuged at 100,000×*g* for 70 min at 4 °C to form pellets containing exosomes. The resulting supernatant was carefully retrieved without disturbing the exosome-containing pellets. Exosome containing pellets were washed with a large volume of ice-cold particle-free PBS. A second round of ultracentrifugation was carried out under the same conditions (100,000×*g* for 70 min at 4 °C). The resulting exosome-containing pellets were resuspended in 100 μl of PBS. Exosomes were examined by electron microscopy using negative staining. Further, exosomes were quantified using a NanoSight NS300 instrument (Malvern Instruments Ltd. UK) equipped with NTA 3.0 analytical software (Malvern Instruments Ltd. UK). Protein concentration in exosomes was estimated using Micro BCA™ Protein Assay Kit (Thermo Fisher Scientific, Waltham, MA, USA).

### Capillary tube formation assay

BD Matrigel™ Basement Membrane Matrix ((#356234, R&D Systems, Minneapolis, MN) was thawed overnight on ice and added to a 48-well plate at 100 µL/well, followed by solidifying for 2 h at 37 °C. HUVECs were trypsinized and suspended in EBM-2 Basal Medium containing EGM-2 SingleQuot Kit Supple & Growth Factor at a density of 1.5 × 10^5^/mL. Subsequently, 200 μL of suspended HUVECs were added to each well, and the slide was incubated for 6 h at 37 °C under a 5% CO_2_ humidified atmosphere. HUVECs were cultured in the presence of 1.5 µg/mL exosomes for 48 h before determination of the role of exosomes in tube formation. Tube formation capacity of the closed networks of vessel-like tubes was analyzed using Image J software.

### Transwell migration assay

Transwell chambers (BD Biosciences, San Jose, CA, USA) were rehydrated and equilibrated for 2 h with 500 µL of serum-free RPMI1640 media. Medium in the inserts was aspirated and inserts were placed into wells containing EBM-2 Basal Medium containing EGM-2 SingleQuot Kit Supple & Growth Factor. HUVECs were trypsinized and suspended in serum-free RPMI1640 media at a density of 5 × 10^5^/mL. Subsequently, 100 μL of suspended HUVECs were added to each well, and the plate was incubated for 12 h at 37 °C under a 5% CO_2_ humidified atmosphere. HUVECs (5 × 10^4^) were cultured in the presence of 0.5 µg exosomes for 48 h to explore the role of exosomes in migration and were then added to each chamber using serum-free RPMI1640 media. Exosomes were incubated in the chambers for 12 h. The medium was then removed and the upper surface of the membrane was scrubbed with a cotton swab. Cells on the lower surface of the scrubbed membranes were fixed using methanol and stained with crystal violet. Images were obtained from five random visual fields in each well, counted and statistically analyzed.

### CCK-8 cytotoxicity assay

Half maximal inhibitory concentration (IC_50_) values in HCC cells subjected to sorafenib were determined using Cell Counting kit-8 (#CK04, CCK-8; Dojindo Laboratories, Japan) according to the manufacture’s instruction. Cells were seeded in 96-well plate at a density of 5000 cells per well and cultured overnight. Further, 10 µL of sorafenib at different concentrations was added to the culture plate and incubated for 48 h at 37 °C. Subsequently, 10 μl of CCK-8 solution was added to each well and then incubated for 2 h at 37 ℃. To determine the role of exosomes in IC_50_ values, every 1 × 10^5^ HCC cells were treated with 1 μg exosomes extracted from corresponding cells for 48 h before the assay. Absorbance (A) of the CCK-8 solution was determined at 450 nm. Relative CCK-8 absorbance was determined based on at least three replicates.

### Small RNA sequencing and data analysis

To prepare the culture medium for exosome small RNA sequencing, GOLPH3 shC and sh1 SNU-449 cells were cultured in DMEM with exosome-free FBS for 48 h. Next, 50 mL of the culture medium was collected from each group, and the collection was repeated three times. Exosome isolation, exosomal RNA extraction, small RNA-sequencing and subsequent analysis were performed at Ribobio Co. Ltd. (Guangzhou, China). Briefly, the culture medium collected from each group were mixed with RiboTM Exosome Isolation Reagent, followed by isolation of the exosome according to the manufacturer’s instructions (Ribobio, China). HiPure Liquid miRNA Kit/HiPure Serum/Plasma miRNA Kit (Megan, China) was then used to extracted exosomal RNA in accordance with the manufacturer’s protocol. The integrity and quantity of exosomal RNA was assessed by Qubit^®^2.0 (Life Technologies, USA) and Agilent 2200 TapeStation (Agilent Technologies, USA), respectively. Fifty ng exosomal RNA of each sample were used to prepare small RNA libraries by NEBNext^®^ Multiplex Small RNA Library Prep Set for Illumina (NEB, USA) according to the manufacturer’s instructions. HiSeq 2500 (Illumina, USA) with single-end 50 bp at Ribobio Co. Ltd. (Ribobio, China) was then used to sequence these libraries. miRDeep2 was used to identify known mature miRNA and predict novel miRNA, whereas Rfam12.1 (www.rfam.xfam.org) and pirnabank (www.pirnabank.ibab.ac.in) were used to identify rRNA, tRNA, snRNA, snoRNA, and piRNA through BLAST. The miRNA expression levels were calculated and normalized by reads per million (RPM) values [RPM  =  (number of reads mapping to miRNA/number of reads in clean data)  × 10^6^]. According to the criteria of |log_2_(Fold Change)|≥ 1 and p value  < 0.05, differential expression between two sets of samples was calculated using edgeR algorithm.

### Luciferase reporter assay

The 3′UTR of PTEN mRNA with the WT and MUT binding sites of miR-494-3p were amplified and cloned downstream to the pSiCheck2 vector (TsingKe Biotech, Beijing, China). Luciferase reporter vector was transfected into 293 T cells in the miR-494-3p mimic, or miR-control. Luciferase activity was determined using the Promega Dual-Luciferase^®^ Reporter Assay System (#E1910; Promega, WI, USA) after transfection for 48 h according to the manufacturer’s instructions. All experiments were performed independently in triplicate.

### Mimic, inhibitor and transient transfection

miR-494-3p mimic and miR-494-3p inhibitor were purchased from Ribobio company (GuangZhou, China). Oligonucleotide transfection was performed using lipofectamine 2000 reagent (Invitrogen, Carlsbad, CA), according to the manufacturer’s instructions. The final concentration of miR-494-3p mimic and miRNA mimic negative control transfected into HUVEC and SNU449 cells was 50 nM. The final concentration of miR-494-3p inhibitor and miRNA inhibitor negative control used was 100 nM.

### Bioinformatic prediction

TargetScan, miRDB, miRTarBase and miRWalk tools were used to predict the potential target genes of miR-494-3p. The intersection gene list predicted by these algorithms was obtained through Venn diagram analysis.

### Statistical analysis

Data were analyzed using GraphPad Prism (Version 8.0) software. Data were expressed as mean  ±  SD from three independent experiments. Differences between two groups were analyzed by two-tailed Student’s *t* tests. Comparison of more than two groups was performed by one-way ANOVA with Dunnett’s multiple comparisons test. Groups with two variables were compared with two-way ANOVA with Tukey’s multiple comparisons test. *p*  < 0.05 was considered statistically significant. No samples or mice were excluded from the analysis as outliers. Mice were randomly allocated to groups using the random number table method. Sample size estimation tests were not conducted for the animal studies.

## Results

### GOLPH3 downregulation suppresses angiogenesis and increases sorafenib sensitivity of HCC cells in vivo.

**Fig. 1:**
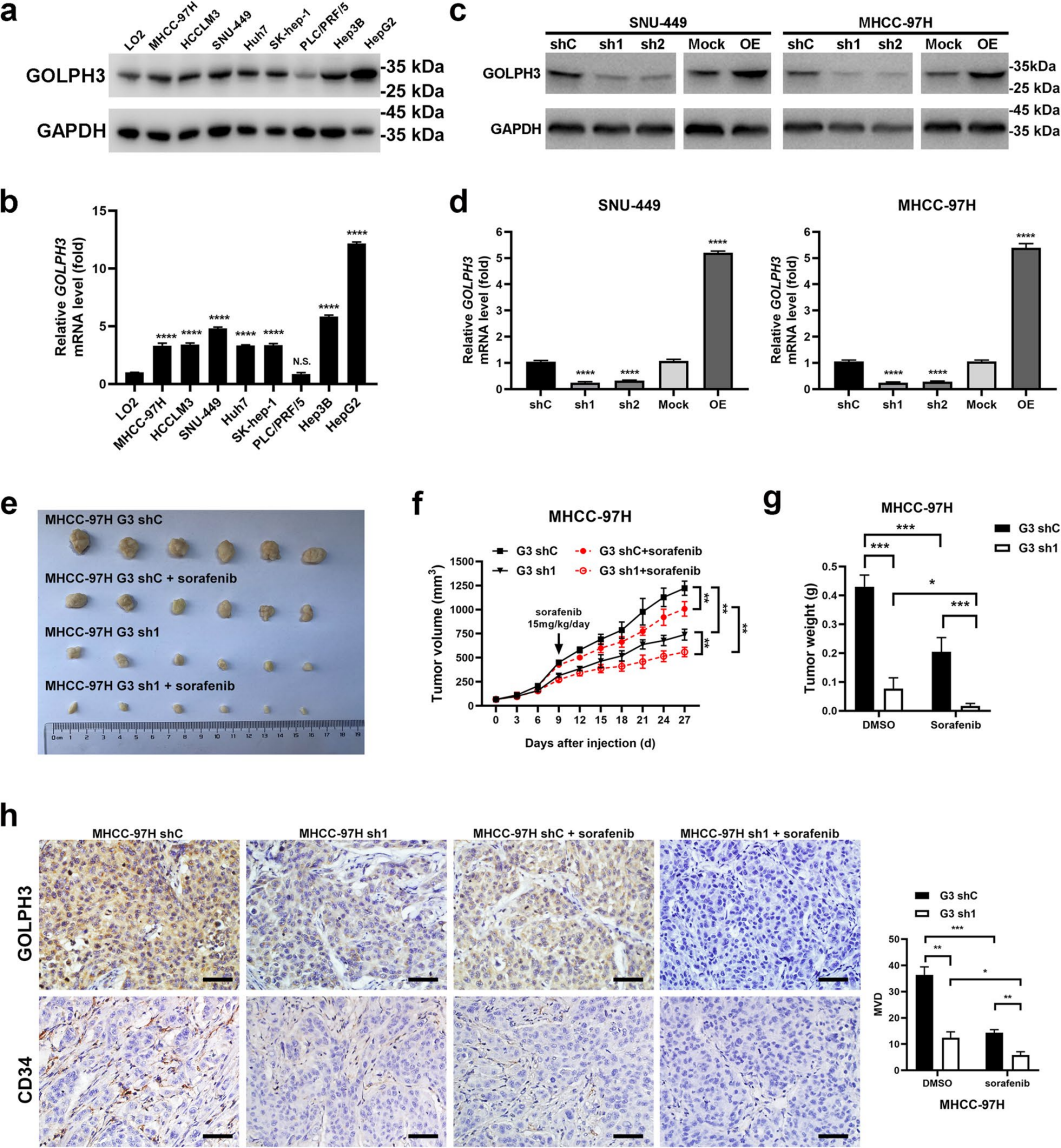
GOLPH3 downregulation suppresses angiogenesis and increases resistance of HCC cells to sorafenib in vivo. **a**, **b** Western blotting and quantitative RT-PCR (qRT-PCR) assay of GOLPH3 expression in LO2 and 8 HCC cell lines. GAPDH was used as a loading control. **c**, **d** Upregulation and downregulation of GOLPH3 in SNU-449 and MHCC-97H cells as confirmed by WB and qRT-PCR assay. GAPDH was used as a loading control. **e** MHCC-97H-shC and MHCC-97H-sh1 cells (1 × 10^7^) were injected subcutaneously into the flanks of nude mice. Mice were intraperitoneally administered with sorafenib or DMSO from day 9 after administration of cells. **f** Tumor volume was determined after every 3 days for all groups and presented as growth curves. **g** Tumor weight was determined and compared for all groups. **h** Immunohistochemistry staining assay of GOLPH3 and CD34 expression in tumors from 2 mice in each group. Number of MVD was calculated from five random fields. Data are expressed as mean  ±  SD and analyzed by one-way ANOVA with Dunnett’s multiple comparisons test (**b**, **d**) or two-way ANOVA with Tukey’s multiple comparisons test (**f**–**h**). *p  < 0.05; **p  < 0.01; ***p  <  0.001; ****p  < 0.0001; *NS* not significant

Eight HCC cell lines were used to explore the expression of GOLPH3 in HCC cells and appropriate cell lines were selected for further investigation. GOLPH3 mRNA and protein levels were analyzed by real-time quantitative (RT-qPCR) and western blot (WB). The results showed that GOLPH3 was overexpressed in seven HCC cell lines (MHCC-97H, HCC-LM3, SNU-449, Huh7, SK-hep-1, Hep3B and HepG2), whereas the human immortalized hepatocyte cell line LO2 and one HCC line PLC/PRF/5 had lower levels of GOLPH3 (Fig. 1a, b). MHCC-97H and SNU-449 were selected for subsequent experiments. GOLPH3 stable overexpression or knockdown MHCC-97H and SNU-449 cell lines were constructed (Fig. 1c, d). Xenograft tumor model in nude mice were generated using MHCC-97H cell lines ectopically expressing GOLPH3 shRNA or control cells to explore the effects of GOLPH3 on tumor growth and sorafenib resistance in vivo (Fig. 1e). Tumor volume and weight decreased in GOLPH3 knockdown group and sorafenib-treated groups compared with the control group (Fig. 1f, g). Moreover, the average tumor volume and weight were significantly smaller in GOLPH3 knockdown plus sorafenib-treated group compared with that of the control group. Anti-CD34 immunohistochemical staining showed that microvessel density (MVD) was lower in the sorafenib treated group, especially in GOLPH3 knockdown plus sorafenib-treated compared with that of the control group (Fig. 1h). These results indicated that GOLPH3 downregulation attenuates HCC angiogenesis and enhances sensitivity to sorafenib in vivo.

### GOLPH3 enhances angiogenesis through exosome activity

**Fig. 2:**
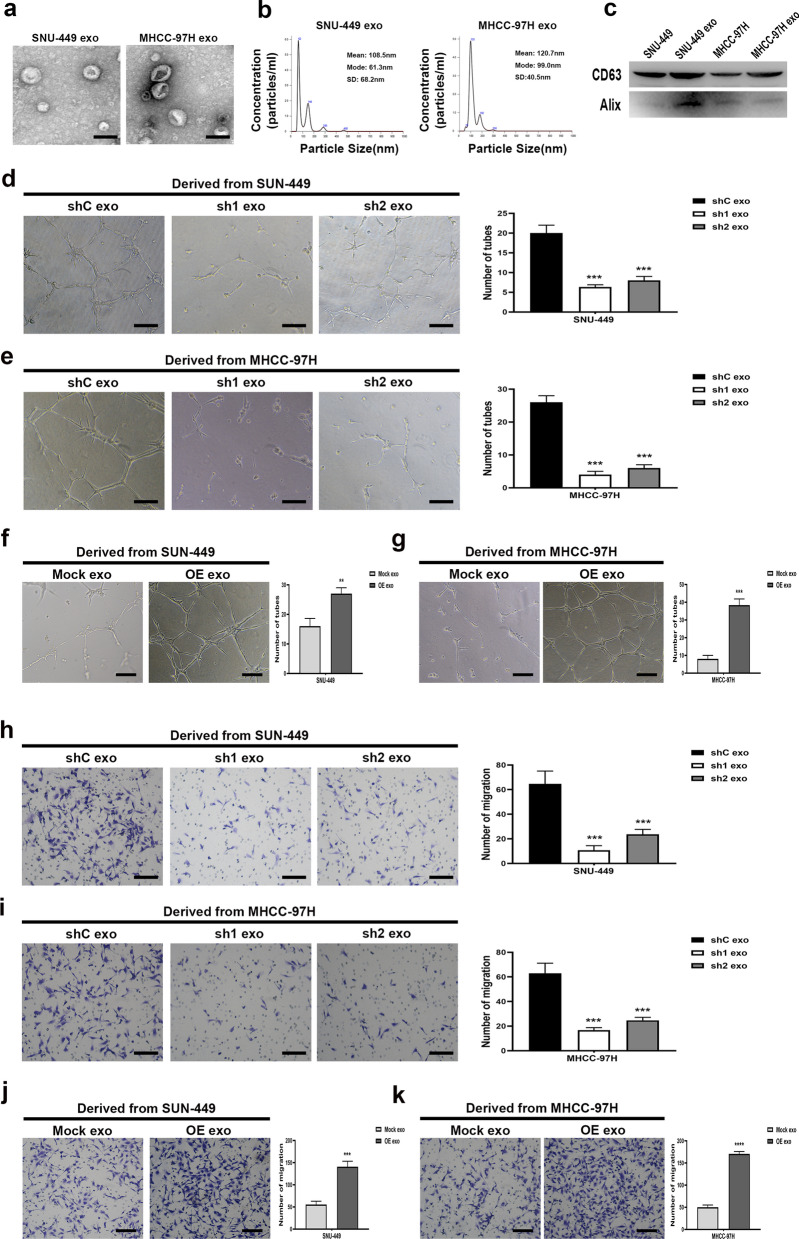
GOLPH3 enhances angiogenesis through exosomes in HUVEC model. **a** Representative images of exosomes obtained by transmission electron microscopy (TEM). **b** Size distribution of exosomes analyzed by NanoSight. **c** Expression levels of exosome markers: CD63 and Alix as determined by western blot analysis. **d**, **e** Tube formation assay of HUVECs treated with exosomes derived from GOLPH3-downregulated HCC cells compared with vector cells. (**f** and **g**) Capillary tube formation assay of HUVECs treated with exosomes derived from GOLPH3-overexpression HCC cells compared with control cells. **h**, **i** Migration rate of HUVECs treated with exosomes as determined by transwell migration assay. **j**, **k** Migration rate of HUVECs treated with exosomes as determined by transwell migration assay. Data are expressed as mean  ±  SD of 3 independent experiments and analyzed by one-way ANOVA with Dunnett’s multiple comparisons test (**d**, **e**, **h**, **i**), or *t *test (**f**, **g**, **j**, **k**). ***p  < 0.001; *Exo* exosome

Exosomes were isolated from the culture medium of HCC cells by differential centrifugation methods to explore whether GOLPH3 promotes HCC angiogenesis and sorafenib resistance through exosome activity. Isolated exosomes were analyzed by transmission electron microscopy (TEM) and presented a cup-shaped morphology or biconcave-disk shape (Fig. 2a). Size distribution of exosomes was quantified by Nanosight analysis. Results from NTA revealed that exosomes extracted from SNU-449 and MHCC-97H groups exhibited similar size distribution (Fig. 2b). Furthermore, western blotting analysis showed that isolated exosomes expressed two important exosome markers, CD63 and Alix (Fig. 2c).

Analysis using in vitro human umbilical vein endothelial cell (HUVEC) model showed that exosomes derived from GOLPH3 knockdown SNU-449 and MHCC-97H cells significantly reduced tube formation and migration of HUVECs compared with exosomes from control cells (Fig. 2d, e, h, i). Exosomes derived from GOLPH3 overexpression SNU-449 and MHCC-97H cells significantly enhanced tube formation and migration of HUVECs (Fig. 2f, g, j, k). These findings indicate that GOLPH3 exerts significant angiogenic effect on HCC cells by through exosome activity.

### Exosomes derived from GOLPH3-overexpressed cells enhances sorafenib resistance in HCC cells

**Fig. 3:**
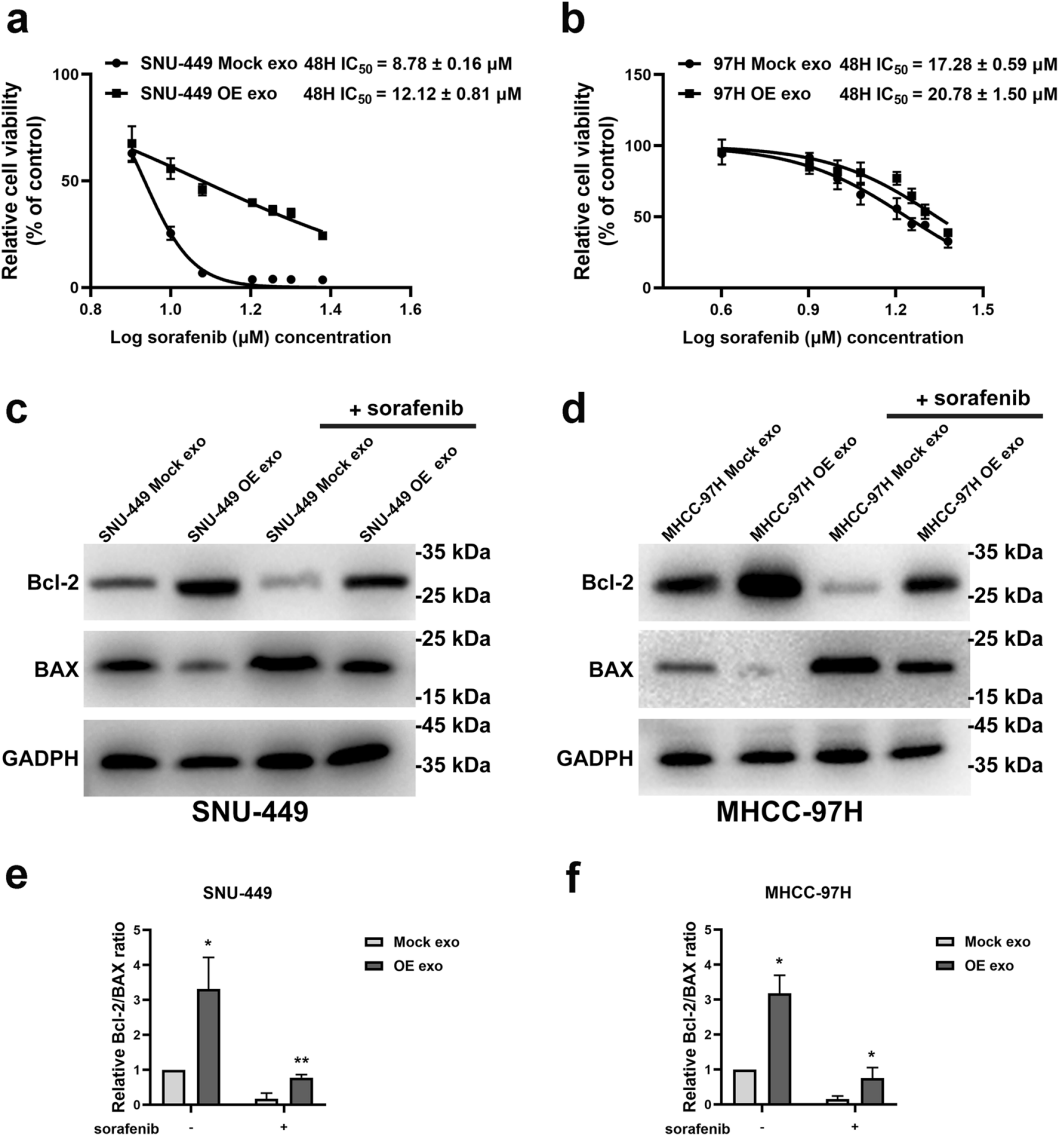
Exosomes from GOLPH3-overexpressed cells enhanced sorafenib resistance in HCC cells. **a** IC_50_ value of SNU-449 treated with indicated exosomes from 3 independent experiments. **b** IC_50_ value of MHCC-97H treated with indicated exosomes from 3 independent experiments. **c** For SNU-449 cells incubated with indicated exosomes and thereafter treated with sorafenib (10 μmol/L) for 48 h, Bcl-2 and Bax levels were determined by western blot, with GAPDH as the normalized control. **d** For MHCC-97H cells incubated with indicated exosomes and exposed to 20 μmol/L sorafenib for 48 h, levels of Bcl-2 and Bax were detected via western blotting. **e**, **f** Grayscale value analysis of Bcl-2/Bax ratio by Image J. Error bars represent mean  ±  SD from 3 independent experiments. Data are analyzed by *t *test (**a**, **b**) or two-way ANOVA with Tukey’s multiple comparisons test (**e**, **f**). *p  < 0.05; **p  < 0.01

SNU-449 and MHCC-97H were incubated with exosomes extracted from GOLPH3-overexpressed HCC cells for 48 h to further explore the effect of exosomes derived from GOLPH3-overexpressed cells on sorafenib resistance. The variation in the half maximal inhibitory concentration (IC_50_) value in HCC cells subjected to sorafenib were explored by CCK-8 cytotoxicity assays. The results showed that SNU-449 and MHCC-97H cells treated with exosomes derived from GOLPH3-overexpressed MHCC-97H cells had higher IC_50_ compared with that of control groups (Fig. 3a, b). These results implied that exosomes generated by GOLPH3-overexpressed cells significantly increased drug resistance to sorafenib-induced growth inhibition.

Bcl-2 was upregulated and Bax was downregulated in HCC cells after incubation with exosomes generated by GOLPH3-overexpressed cells, with or without sorafenib treatment (Fig. 3c, d). Bcl-2 is an anti-apoptotic protein whereas Bax enhances apoptosis, thus increased Bcl-2/Bax ratio is a marker of apoptosis inhibition [20, 21]. Increase in the Bcl-2/Bax ratio was correlated with the anti-apoptosis activity induced by exosomes generated by GOLPH3-overexpressed HCC cells (Fig. 3e, f). These results confirmed that exosomes generated by GOLPH3-overexpressed HCC cells promoted sorafenib resistance in HCC cells by inhibiting sorafenib-induced apoptosis.

### Exosomal miR-494-3p expression level was correlated with GOLPH3 expression level in HCC

**Fig. 4:**
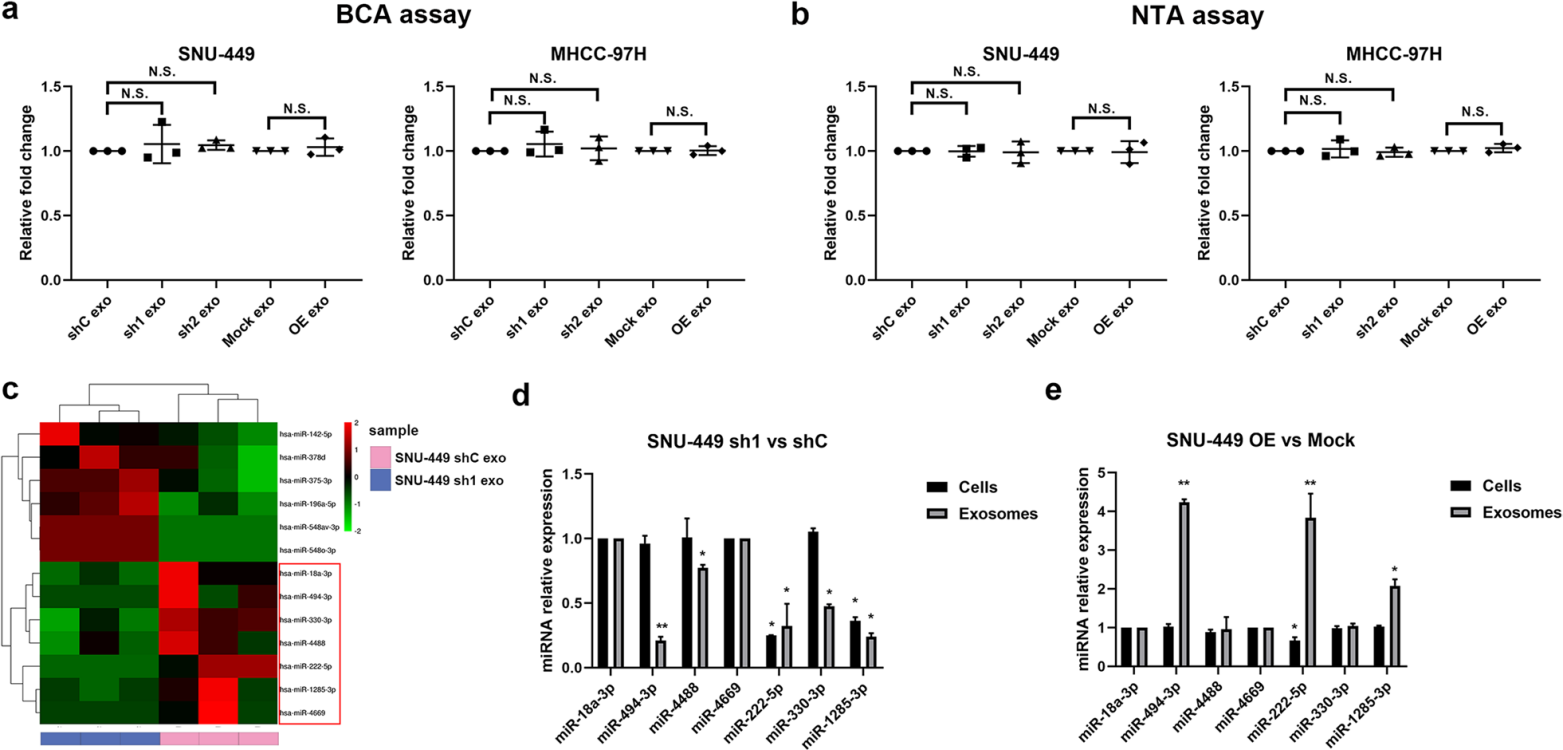
Exosomal miR-494-3p is correlated with GOLPH3 expression. Relative fold changes in protein concentrations of exosomal lysates as examined by the BCA assay (**a**) and NanoSight (**b**). **c** Heatmap for differential miRNAs in exosomes between SUN-449 shC and sh1 group. **d**, **e** Expressions of differential miRNAs in GOLPH3-downregulated or GOLPH3-overexpressed SNU-449 cells and their corresponding exosomes were validated by qRT-PCR. All experiments were repeated at least 3 times. Error bars represent mean  ±  SD for 3 independent experiments. Data are analyzed by one-way ANOVA with Dunnett’s multiple comparisons test (**a**, **b**) or *t *test (**c**, **d**, **e**). *p  < 0.05; **p  < 0.01; *NS* not significant

Further analysis was conducted to explore the mechanisms underlying promotion of angiogenesis and sorafenib resistance by GOLPH3 through exosomes. Protein concentrations of the exosome lysates in the vector and GOLPH3 knockdown groups were determined. The results showed that protein concentrations in GOLPH3-overexpressed group were not significantly different compared with those of the control group (Fig. 4a); NanoSight analysis also indicated that GOLPH3 did not affect the quantity of proteins in the exosome (Fig. 4b).

Tumor-derived exosomes promoted angiogenesis and drug resistance of tumor cells by carrying and delivering signaling molecules, especially miRNAs [22]. Thus, we hypothesized that GOLPH3 regulated the exosomal miRNA content to promote angiogenesis and sorafenib resistance. Small RNA sequencing (sRNA-seq) was performed to explore expression of miRNAs altered by GOLPH3 in exosomes derived from GOLPH3-downregulated SNU-449 cells. Analysis of the miRNA profile revealed that expression of hsa-miR-18a-3p, hsa-miR-494-3p, hsa-miR-330-3p, hsa-miR-4488, hsa-miR-222-5p, hsa-miR-1285-3p and hsa-miR-4669 levels were downregulated in exosomes derived from GOLPH3 knockdown cells compared with the control (Fig. 4c). Notably, qRT-PCR experiments showed that miR-494-3p expression levels were positively correlated with GOLPH3 levels in exosomes, whereas cellular total miR-494-3p expression levels were unaltered (Fig. 4d, e). These results implied that GOLPH3 increased miR-494-3p content in exosomes and did not induce transcription of miR-494-3p in HCC cells.

### Exosomal miR-494-3p modulates HUVECs angiogenesis

**Fig. 5:**
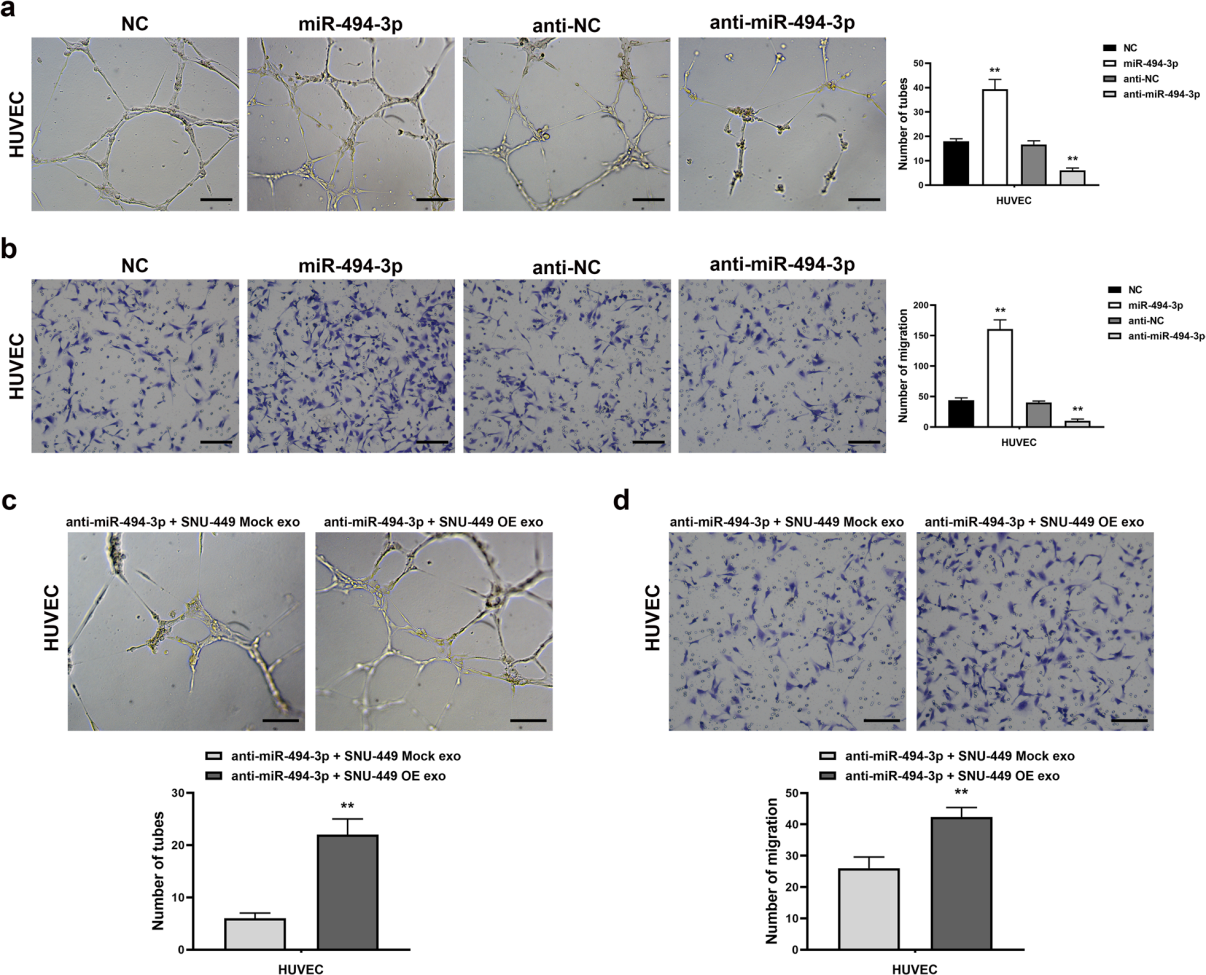
Exosomal miR-494-3p regulated HUVEC angiogenesis. Abilities of HUVECs capillary tube formation (**a**) and migration (**b**) in vitro were largely strengthened in miR-494-3p-overexpressing, while potentially compromised in miR-494-3p-silenced. Capacities for in vitro capillary tube formation (**c**) and migration (**d**) by HUVECs was partly reversed by exosomes derived from GOLPH3-overexpressing SNU-449 cells after miR-494-3p knockdown. Error bars represent mean  ±  SD from 3 independent experiments. Data are analyzed by *t *test **a**–**d**. **p  < 0.01

miR-494-3p mimics were transfected into HUVECs to upregulate miR-494-3p expression to explore whether GOLPH3 modulates angiogenesis through activity of exosomal miR-494-3p. The results showed that the upregulation of miR-494-3p expression increased migration rate and capillary tube formation ability of HUVECs (Fig. 5a, b). Conversely, miR-494-3p inhibitor substantially decreased the migration rate and tube formation ability of HUVECs (Fig. 5a, b). Notably, the effects of miR-494-3p inhibitor were partly rescued by administration of exosomes derived from GOLPH3-overexpressed SNU-449 cells (Fig. 5c, d). These findings indicate that increased expression of miR-494-3p in exosome mediates GOLPH3 induced HCC cell angiogenesis.

### Exosomal miR-494-3p regulates sorafenib resistance of HCC cells

**Fig. 6:**
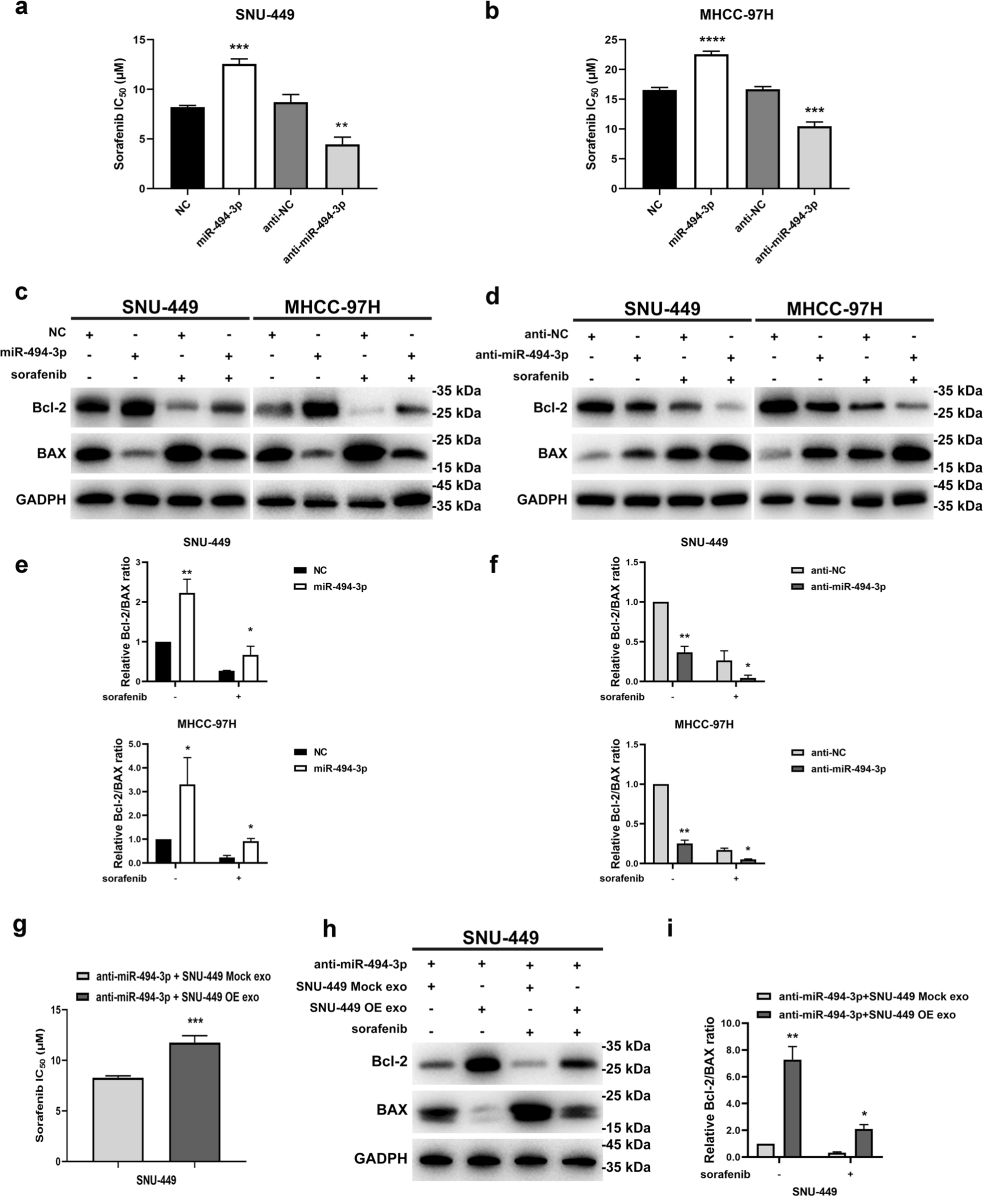
Exosomal miR-494-3p regulates sorafenib resistance in HCC cells. **a**, **b** IC_50_ value were decreased in miR-494-3p-silenced, and increased in miR-494-3p-overexpressing HCC cells. **c** SNU-449-NC, SNU-449-miR-494-3p, SNU-449-anti-NC and SNU-449-anti-miR494-3p cells were treated with sorafenib (10 μmol/L) for 48 h, then, treated cells were harvested to detect Bcl-2 and Bax levels. GAPDH was used as the loading control. **d** MHCC-97H cells transfected with NC, miR-494-3p mimics, anti-NC and miR-494-3p inhibitor, respectively were exposed to 20 μmol/L sorafenib for 48 h, and Bcl-2 and Bax levels determined by WB. **e**, **f** Histogram for the ratio of relative gray values of Bcl-2 and Bax proteins in **c** and **d**. **g** The decrease in IC_50_ value in SNU-449 cells transfected with the miR-494-3p inhibitor was partly reversed by exosomes derived from GOLPH3-overexpressed SNU-449 cells. **h** For SNU-449 cells transfected with the anti-NC or miR-494-3p inhibitor incubated with indicated exosomes and treated with sorafenib (10 μmol/L) for 48 h, Bcl-2 and Bax levels in indicated cells were determined by western blot, with GAPDH as the normalized control. **i** The histogram describes the Bcl-2/Bax ratios in** h** by gray value analysis. Data are expressed as mean  ±  SD from 3 independent experiments and analyzed by *t* test (**a**, **b, g**) or two-way ANOVA with Tukey’s multiple comparisons test (**e**, **f**, **i**). *p  < 0.05; **p  < 0.01; ***p  < 0.001

CCK-8 assays and determination of IC_50_ were conducted to further explore the effect of exosomal miR-494-3p on sorafenib resistance of HCC cells. Overexpression of miR-494-3p significantly upregulated IC_50_ values in SNU-449 and MHCC-97H cells after sorafenib treatment. Conversely, downregulation of miR-494-3p sensitized HCC cells to sorafenib treatment (Fig. 6a, b). Exosomes generated by GOLPH3-overexpressed HCC cells decreased sensitivity of HCC cells to sorafenib by inhibiting apoptosis, thus miR-494-3p promoted sorafenib resistance by suppressing apoptosis. A significant increase in expression level of Bax was observed in miR-494-3p downregulated cells treated with sorafenib (Fig. 6c, d). Moreover, downregulation of Bcl-2 was observed in SNU-449 cells and MHCC-97 cells after transfection with miR-494-3p inhibitor and/ or treatment with sorafenib for 48 h (Fig. 6c, d). Decrease in Bcl-2/Bax ratio indicated that administration of miR-494-3p inhibitor increased sensitivity of HCC cells to sorafenib by inducing apoptosis (Fig. 6e, f). Notably, upregulation of Bcl-2 and simultaneous downregulation of Bax protein was observed when miR-494-3p was overexpressed in HCC cells with and without sorafenib treatment (Fig. 6c, d). Bcl-2/Bax ration was significantly increased by administration of miR-494-3p mimics indicating that miR-494-3p overexpression confers anti-apoptotic effect (Fig. 6e, f). These results indicated that miR-494-3p is implicated in modulation of sorafenib resistance. A series of rescue experiments demonstrated that sorafenib sensitivity and apoptosis of HCC cells induced by miR-494-3p inhibition was partly reversed by administration of exosomes derived from GOLPH3-overexpressed cells (Fig. 6g–i). In summary, these results indicate that exosomal miR-494-3p plays an important role in mediating the effect of GOLPH3 on sorafenib resistance.

### Exosomal miR-494-3p enhances angiogenesis and sorafenib-resistance by directly targeting PTEN mRNA

**Fig. 7:**
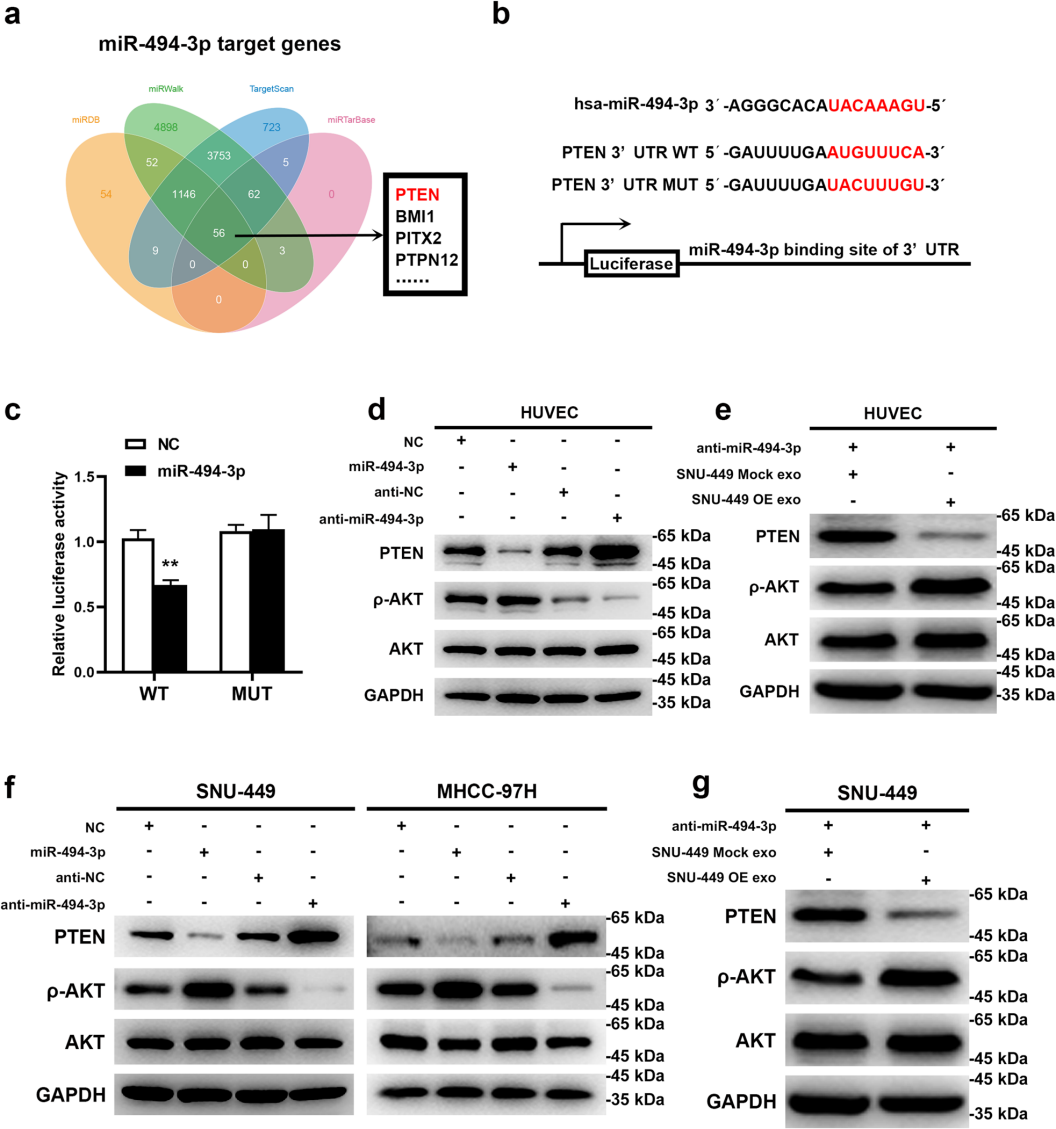
Exosomal miR-494-3p enhanced angiogenesis and sorafenib-resistance by directly targeting PTEN mRNA. **a** Target genes for miR-494-3p were predicted by miRDB, miRWalk, miRanda, and TargetScan; intersection gene lists predicted by these algorithms was analyzed using a Venn diagram. **b** WT and MUT of miR-494-3p binding site on 3′-UTR of PTEN mRNA. **c** miR-494-3p inhibited the activities of the luciferase reporter that contained the wild-type but not the mutant-type 3′UTR of PTEN. **d**, **f** Effects of miR-494-3p overexpression or downregulation on protein levels of PTEN, ρ-AKT and AKT in HUVECs and HCC cells. **e**, **g** After miR-494-3p inhibition, effects of exosomes derived from GOLPH3-overexpressed SNU-449 cells on PTEN, ρ-AKT and AKT levels in HUVEC and SNU-449 HCC cells. Data are expressed as mean  ±  SD for 3 independent experiments and analyzed by *t *test (**c**). **p  < 0.01

PTEN was identified as a tentative target of miR-494-3p through miRNA target prediction (Fig. 7a). Results from luciferase reporter assay indicated that miR-494-3p mimic significantly repressed luciferase activity of 293 T cells transfected with PTEN-3’UTR wild-type (WT) vector, however, the mutant-type (MUT) vector had no effect on the luciferase activity (Fig. 7b, c). Ectopic expression of miR-494-3p downregulated expression of PTEN whereas expression of the downstream target ρ-AKT was upregulated (Fig. 7d, f). On the contrary, inhibition of miR-494-3p upregulated expression of PTEN and downregulated expression of ρ-AKT at the protein level. A rescue experiment demonstrated that exosomes derived from GOLPH3-overexpressed SNU-449 cells alleviated the upregulated expression of PTEN and downregulated expression of ρ-AKT induced by miR-494-3p inhibition in HUVECs and SNU-449 cells (Fig. 7e, g). These results implied that exosomal miR-494-3p enhances angiogenesis and sorafenib resistance by directly inhibiting PTEN protein expression.

## Discussion

Angiogenesis is a characteristic feature of HCC and novel therapies targeting angiogenesis provide promising breakthroughs for HCC treatment [2, 4]. In the past 10 years, sorafenib is used as the first-line drug in the treatment of advanced-stage HCC with its anti-angiogenic effect [4, 6]. However, only 30% of HCC patients benefit from sorafenib and acquired resistance occurs within six months after treatment [5]. Therefore, it is imperative to identify new potential targets or therapeutic strategies to overcome this acquired resistance.

Previous studies report that aberrant expression of GOLPH3 is highly associated with advanced tumor stage and vascular invasion in HCC [9, 13]. Recent studies indicate that GOLPH3 plays important roles in drug resistance in multiple tumors [8, 23, 24]. However, the mechanism of GOLPH3 in modulating sorafenib resistance in HCC has not been explored. In the presents study, GOLPH3 expression was significantly upregulated in HCC cell lines compared with normal hepatocyte cell line which is consistent with findings from previous studies. Findings from in vivo nude mice experiment showed that sorafenib administration and GOLPH3 knockdown decreased tumor growth and angiogenesis independently. Notably, the tumor growth and angiogenesis were further decreased in cells with GOLPH3 knockdown and treated with sorafenib. These findings indicate that GOLPH3 downregulation attenuates HCC progression, angiogenesis and enhances sensitivity of HCC cells to sorafenib.

Exosomes provide a new mechanism for intercellular communication and are implicated in tumorigenesis, tumor metastasis, immune escape, and drug resistance of HCC [25–27]. Therefore, analyses were conducted to explore whether GOLPH3 modulates HCC angiogenesis and sorafenib resistance through an exosomal mechanism. Our findings showed that exosomes derived from GOLPH3-overexpression HCC cells increased the capacity of tube formation and migration rate of HUVECs. Notably, exosomes derived from GOLPH3-overexpressed cells suppressed apoptosis of HCC cells induced by sorafenib treatment and reduced sensitivity of HCC cells to sorafenib. These findings indicated that exosomes generated by GOLPL3 overexpressed cells enhance angiogenesis and resistance of HCC cells to sorafenib.

Moreover, the results showed that GOLPH3 had no effect on the quantity of exosomes. A previous study reported that GOLPH3 did not increase secretion levels of exosomes but affected miRNAs expression profile in GBM cell-derived exosomes [28]. Tumor-derived exosomes contain a substantial number of cancer-related markers, such as miRNAs, which are potential targets for effective treatment of HCC [29, 30]. For example, exosomal miR-122 enhances drug sensitivity and inhibits angiogenesis in HCC [27]. Several studies report that GOLPH3 plays essential roles in Golgi-to-plasma (PM) membrane trafficking implicated in transport of targeting cargoes to extracellular vesicles, thus affecting malignant secretory phenotypes [31, 32]. Therefore, analysis was conducted to explore whether GOLPH3 enhances angiogenesis and sorafenib resistance by affecting the inclusions of exosomes derived from HCC cells. Exosome miRNA sequencing and further validation results showed that GOLPH3 expression levels was significantly correlated with miR-494-3p expression level in exosomes derived from HCC cells. Loading of miRNAs into exosomes mainly depends on two mechanisms: passive enrichment of miRNAs with high expression in cells and a selective sorting mechanism, which is independent of cellular expression level [33, 34]. In the present study, miR-494-3p expression level in cells was not regulated by GOLPH3 overexpression. These findings indicate that GOLPH3 promoted selective sorting of miR-494-3p into exosomes from the cytoplasm of HCC cells. A miRNA motif and sumoylated heterogeneous nuclear ribonucleoproteins (hnRNPs)-dependent pathway are potential modes for sorting of miRNAs to exosomes. In 2013, Villarroya-Beltri et al. observed that sumoylated hnRNPA2B1 recognizes the GGAG motif in the 3′portion of miRNA sequences and promotes packaging of specific miRNAs into exosome [35]. In 2019, Lee et al. reported that oxidative stress induces O-GlcNAcylation of hnRNPA2B1 leading to significant alteration in hnRNPA2B1-bound miRNA repertoire [36]. GOLPH3 is implicated in affecting the retrograde intra-Golgi trafficking of protein glycosyltransferases [10]. These findings imply that GOLPH3 affects binding of miR-494-3p motif to hnRNPs, potentially through glycosylation of hnRNPs. The results indicate that GOLPH3 plays an important regulatory role in sorting of exosomal miRNA. However, whether GOLPH3 promotes the selective sorting of miR-494-3p exosomes through hnRNPs-dependent pathways, and which hnRNA proteins are involved in this process need to be verified by further studies.

**Fig. 8:**
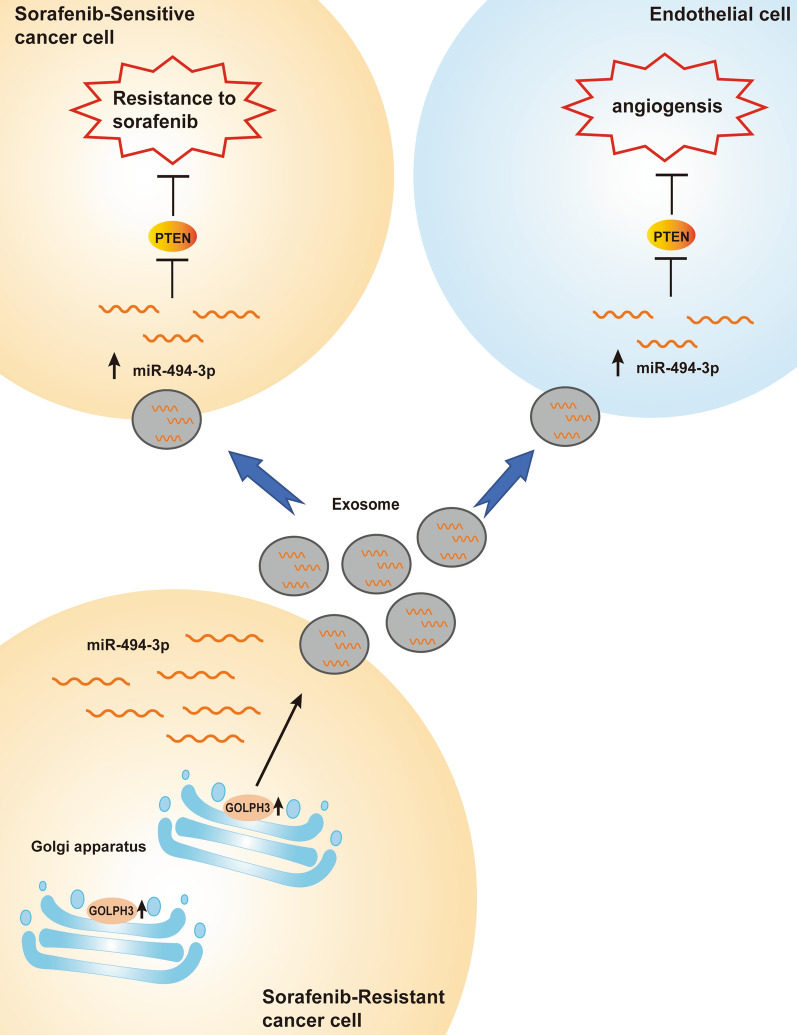
Function and mechanism of GOLPH3 in HCC angiogenesis and sorafenib resistance. GOLPH3 promotes HCC angiogenesis and sorafenib resistance by enhancing exosomal miR-494-3p secretion to recipient HUVECs and HCC cells, respectively

miR-494-3p is a tumor-derived miRNA and its expression is significantly upregulated in multiple types of cancer. miR-494-3p enhances core hallmarks of cancer by targeting several important tumor suppressor genes, such as PTEN, SOCS6 and BMI1 [37–40]. PTEN is implicated in suppression of angiogenesis and drug resistance by inactivating AKT pathway [41]. The findings of the present study indicated that miR-494-3p promoted angiogenesis and sorafenib resistance by targeting PTEN. Moreover, the findings showed that inhibition of downregulation of PTEN expression induced by miR-494-3p was partly reversed by administration of exosomes derived from GOLPH3-overexpressed SNU-449 cells. These studies demonstrate that GOLPH3 promotes HCC angiogenesis and sorafenib resistance by enhancing exosomal miR-494-3p secretion to recipient HUVECs and HCC cells, respectively (Fig. 8). However, in vivo experiments are required to further verify the effect of exosomal miR-494-3p in HCC angiogenesis and sorafenib resistance. Moreover, in the protection of phospholipid bilayer membrane, exosomal miRNA is resistance to degradation, implying that exosomal miRNAs are potentially novel markers for HCC. Large sample clinical studies are required to investigate the clinical significance of exosomal miR-494-3p as a prognostic factor for HCC patients.

### Conclusion

In summary, the findings of the present study indicate that GOLPH3 enhances angiogenesis and sorafenib resistance by modulating miR-494-3p expression in HCC cells derived-exosomes. Higher expression level of miR-494-3p in exosomes suppressed PTEN protein expression by directly targeting the 3′UTR of PTEN mRNA. Moreover, the results indicated that targeting GOLPH3 and exosomal miR-494-3p has important potential clinical value in improving therapeutic efficiency in HCC patients.

## Data Availability

The datasets used or analyzed during the current study are available from the corresponding author on reasonable request.

## Abbreviations

G3: GOLPH3, Golgi phosphoprotein 3
HCC: Hepatocellular carcinoma
NTA: Nanoparticle tracking analysis
WB: Western blot
TEM: Transmission electron microscopy
sRNA-seq: Small RNA sequencing
RT-qPCR: Real-time quantitative
MVD: Microvessel density
HUVEC: Human umbilical vein endothelial cell
IC_50_: Half maximal inhibitory concentration
WT: Wild-type
MUT: Mutant-type
TGN: Trans-Golgi network
VEGF: Vascular endothelial growth factor
hnRNPs: Heterogeneous nuclear ribonucleoproteins
